# Chitosan: Structural and Chemical Modification, Properties, and Application

**DOI:** 10.3390/ijms25010554

**Published:** 2023-12-31

**Authors:** Joanna Kluczka

**Affiliations:** Department of Inorganic Chemistry, Analytical Chemistry and Electrochemistry, Silesian University of Technology, B. Krzywoustego 6, 44-100 Gliwice, Poland; joanna.kluczka@polsl.pl

## 1. Introduction

Chitosan is a polymer of natural origins that possesses many favourable properties. It can form chelates, is non-toxic, biocompatible with the human body, and biodegradable. These and more properties make chitosan a versatile material. Furthermore, it can be used to produce physical forms, such as hydrogels, nanoparticles, pastes, nanofibers, films, membranes, microgranules, sponges, etc. By modifying chitosan through crosslinking, grafting, combining it with other materials, or ion templating, various targeted properties of this polymer can be obtained, resulting in new and specific applications.

**Figure 1:**
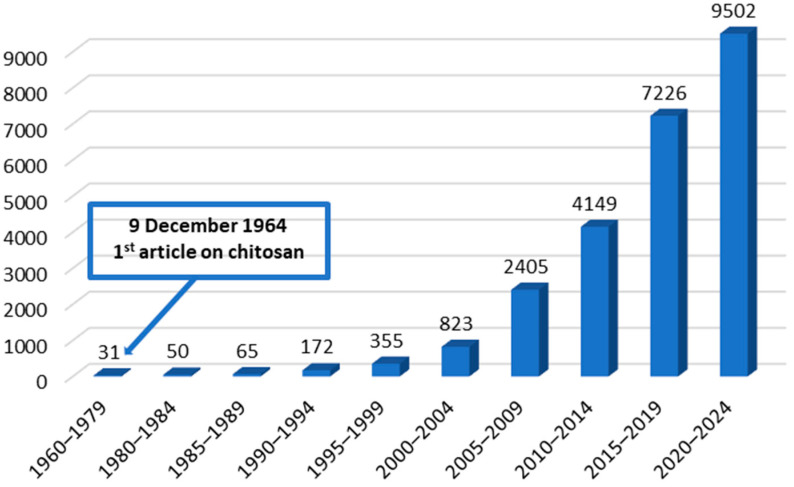
Numbers of papers on chitosan from the outset until 15 December 2023. Source: own elaboration based on the Science Direct database [1].

The first scientific article on chitosan in the ScienceDirect database appeared on 9 December 1964 (Figure 1). It concerns yeast fermentation in bakery dough using chitosan as a fermentation inhibitor [1]. Subsequent works mainly concerned the sorption properties of chitosan and its use in chromatographic methods of concentrating metals; later studies focus on dyes present in liquids such as organic solutions, fresh water, and salt water [2]. It was discovered that chitosan has many interesting and useful properties. The physical and chemical properties, hydrolysis, and the degree of deacetylation of chitosan have been studied [3,4,5]. In the 1990s, there was a huge increase in interest in chitosan, which resulted in a several-fold increase in the number of articles on chitosan compared to the 1980s. Topics related to the modification and crosslinking of chitosan were discussed, for example, the possibility of using its mucoadhesive properties in pharmaceutical technology [6,7].

Research on the structures, properties and applications of chitosan is continually growing. In Figure 1, the number of articles on chitosan (articles in the ScienceDirect database with the keyword “chitosan” in the title) can be seen; in the subsequent five-year periods of the latter half of the 21st century, we observe an approximately two-fold increase in articles. From the beginning of 2023 until mid-December 2023, as many as 2922 papers with the word “chitosan” in the title were published.

The new Special Issue, entitled “Chitosan: Structural and Chemical Modification, Properties and Application”, of the International Journal of Molecular Sciences includes a total of seven contributions: five original articles and two reviews providing new information about the properties and uses of various physical forms and modifications of chitosan.

One paper [8] presents an overview of methods for preparing chitosan-derived porous materials, and discusses their potential applications. This class of materials has attracted significant attention due to their biocompatibility, nontoxicity, antibacterial properties, and biodegradability, which make them advantageous in various applications. This review discusses five strategies for fabricating porous chitosan materials, pointing out that the fabrication method influences the structure of the obtained material and the potential applications of the considered materials. Porous chitosan scaffolds obtained using a cryogelation method are used in cell culture applications [9] and bone tissue engineering [10]; materials such as scaffolds [11,12,13], microspheres [14,15,16], and membranes [17,18] prepared by freeze-drying are popular in tissue engineering [19,20,21]. They are also utilised in the pharmaceutical industry to control drug release [22], and used to remove ions from water [16]. Finally, they are used in heterogeneous catalysis [18], and chitosan aerogels obtained by the sol-gel method have a high specific surface area, resulting in a great capacity for the adsorption of dyes from water [23].

The properties of chitosan are influenced by its molar mass and degree of deacetylation (DD) [24]. There are various methods available for determining the DD of chitosan, such as elemental analysis [25], conductometric titration [26], potentiometric titration [27], proton nuclear magnetic resonance (^1^H NMR) [28], carbon-13 (^13^C) NMR [29], infrared spectroscopy [30], X-ray diffraction [31], thermal analysis [32], gas chromatography [33], nitrite deamination [34], and a novel detection method—ultrahigh-performance liquid chromatography/mass spectrometry (UPLC–MS/MS) [24]. It has been found that the DD of chitosan affects both the number of free amino groups and the polymer chain conformation, which in turn is related to the mucoadhesive properties of chitosan [35,36]. Chitosan is a commonly used mucoadhesive polymer for delivering drugs nasally; such drugs may include insulin, vaccines, pain medications, peptides, and drugs for neurological disorders [37]. Recently, there has been greater focus on developing chitosan nano-delivery systems for nose-to-brain applications, including hydrogels, emulsions, and nanoparticles (NPs) [38,39,40]. One such method uses a 2,6-pyridine dicarboxylic acid as a biocompatible crosslinker to obtain stable and cytocompatible crosslinked chitosan nanoparticles. Gagliardi et al. [35] found that these CHI-DA-NPs had optimal physicochemical and biological features, making them suitable for future applications as an intranasal delivery system for the brain.

As proven in many studies, chitosan is a polymer that can be used to produce hydrogels suitable for the 3D printing of customized drug delivery systems [41]. Various modifications are used to increase the printability of chitosan hydrogels. Cardoso et al. [42] researched this phenomenon, and discovered that in adding starch to the chitosan hydrogel, its structural characteristics were significantly improved. The hydrogel produced in this manner was then found to be appropriate for creating scaffolds with an extrusion-based 3D bioprinter. Said chitosan–starch scaffolds were found to have a well-defined pore structure that could be utilized to integrate an active component into the matrix, or load it into the pores.

Chitosan’s biocompatibility and the film-forming properties make it a suitable candidate for the development of protective coatings for metal substrates, and a possible alternative to the toxic commercial products that are currently used by conservators. In the field of metal protection, chitosan has been proposed as a coating for industrial steel [43,44], copper [45], aluminium [46], silver [47], and modern bronze alloys [48]. Silver, when exposed to air, tarnishes, darkening in colour and losing shine. It can be protected by a chitosan-based coating, which is a green alternative to commercial acrylic resins, does not require the use of toxic solvents, and does not produce waste [47].

Many chemical modifications of the chitosan macromolecule are currently under investigation to further improve adsorption properties. Hydrogel beads are the most suitable form of chitosan for the purpose of the adsorption process. A significant advantage of using chitosan hydrogels as adsorbents in the natural environment problems is their non-toxicity and biodegradability. An example is the adsorption removal of phosphates from water and wastewater [49,50]. Different surface modifications can be employed by adding active fillings like metal oxides and hydroxides, carbons and biochars, zeolites and minerals, magnetic nanoparticles, and others to the chitosan matrix to enhance the capability of chitosan towards phosphate anions or other ions [51,52]. The effectiveness of magnetic-filled chitosan microspheres (MRCM) in removing dyes from real solutions has been studied through reversed-phase suspension crosslinking polymerization. According to Yu et al. [53], MRCM is a suitable adsorbent for removing methyl orange from water, considering its physical and chemical properties and adsorption thermodynamics. Moreover, MRCM has the potential to be developed as a sorbent for removing other cationic dyes similar to methyl orange from water, as it is an environmentally friendly and reusable sorbent that can be regenerated.

The number of studies on green methods for the synthesis of chitosan-derived Schiff bases has increased. Green synthesis methods minimize the use of hazardous chemicals and reduce the impact of chemical processes on the environment [54].

As current scientific reviews and books show, intensive research on the properties of chitosan is still ongoing, and recent years have brought about great interest in chitosan from two particular perspectives: drug delivery (i.e., the design of modern drugs) [55,56] and packaging systems [57,58]. Chitosan-based films are a promising innovation in active food packaging, as they possess antioxidant and antimicrobial properties. These films have gained recognition for their ability to prolong the shelf-life of perishable food items. Chitosan coatings are effective in preserving fruits and vegetables and maintaining the high-quality characteristics of fresh products [59]. Other modern uses of chitosan include tissue regeneration and wound healing, agriculture, food processing, wastewater treatment, industrial papermaking, cosmetics, textile, photography, bio-refinery work, healthcare, and others [60].

The aforementioned articles indicate that the uses of chitosan are expanding. This is because researchers are improving its existing properties, all the while discovering new ones. Due to the variety of physical forms that chitosan can take, and the modifications that we can make, it is being applied in a multitude of new areas.
